# Overall Survival Prediction of Glioma Patients With Multiregional Radiomics

**DOI:** 10.3389/fnins.2022.911065

**Published:** 2022-07-07

**Authors:** Asma Shaheen, Syed Talha Bukhari, Maria Nadeem, Stefano Burigat, Ulas Bagci, Hassan Mohy-ud-Din

**Affiliations:** ^1^Department of Mathematics, Computer Science and Physics, University of Udine, Udine, Italy; ^2^Department of Electrical Engineering, School of Science and Engineering, Lahore University of Management Sciences, Lahore, Pakistan; ^3^Department of Radiology, Feinberg School of Medicine, Northwestern University, Chicago, IL, United States; ^4^Department of Biomedical Engineering, Northwestern University, Chicago, IL, United States; ^5^Department of Electrical and Computer Engineering, Northwestern University, Chicago, IL, United States

**Keywords:** glioblastoma, radiomics, brain tumor segmentation, survival prediction, machine learning, deep learning, MRI

## Abstract

Radiomics-guided prediction of overall survival (OS) in brain gliomas is seen as a significant problem in Neuro-oncology. The ultimate goal is to develop a robust MRI-based approach (i.e., a radiomics model) that can accurately classify a novel subject as a short-term survivor, a medium-term survivor, or a long-term survivor. The BraTS 2020 challenge provides radiological imaging and clinical data (*178* subjects) to develop and validate radiomics-based methods for OS classification in brain gliomas. In this study, we empirically evaluated the efficacy of four multiregional radiomic models, for OS classification, and quantified the robustness of predictions to variations in automatic segmentation of brain tumor volume. More specifically, we evaluated four radiomic models, namely, the Whole Tumor (*WT*) radiomics model, the *3-subregions* radiomics model, the *6-subregions* radiomics model, and the *21-subregions* radiomics model. The *3-subregions* radiomics model is based on a physiological segmentation of whole tumor volume (WT) into three non-overlapping subregions. The *6-subregions* and *21-subregions* radiomic models are based on an anatomical segmentation of the brain tumor into *6* and *21* anatomical regions, respectively. Moreover, we employed six segmentation schemes – five CNNs and one STAPLE-fusion method – to quantify the robustness of radiomic models. Our experiments revealed that the *3-subregions* radiomics model had the best predictive performance (mean AUC = 0.73) but poor robustness (RSD = 1.99) and the *6-subregions* and *21-subregions* radiomics models were more robust (RSD  1.39) with lower predictive performance (mean AUC  0.71). The poor robustness of the *3-subregions* radiomics model was associated with highly variable and inferior segmentation of tumor core and active tumor subregions as quantified by the Hausdorff distance metric (4.4−6.5mm) across six segmentation schemes. Failure analysis revealed that the *WT* radiomics model, the *6-subregions* radiomics model, and the *21-subregions* radiomics model failed for the same subjects which is attributed to the common requirement of accurate segmentation of the WT volume. Moreover, short-term survivors were largely misclassified by the radiomic models and had large segmentation errors (average Hausdorff distance of 7.09mm). Lastly, we concluded that while STAPLE-fusion can reduce segmentation errors, it is not a solution to learning accurate and robust radiomic models.

## Introduction

Gliomas are brain tumors that originate in the glial cells of the brain (Cha, 2006). They constitute 80% of malignant brain tumors (Goodenberger and Jenkins, 2012). Based on the aggressiveness of the tumor, World Health Organization (WHO) classified them into four grades (Louis et al., 2016): WHO Grade I and Grade II gliomas are called low-grade gliomas (LGGs) and WHO Grade III and Grade IV gliomas are called high-grade gliomas (HGGs). Compared to LGGs, HGGs are more aggressive and malignant with a median survival of less than 2 years (Ohgaki and Kleihues, 2005; Louis et al., 2007; Bi and Beroukhim, 2014). Diagnostic and prognostic evaluations of these tumors are employed using magnetic resonance imaging (MRI) techniques. MRI is a noninvasive imaging modality that is routinely used for three-dimensional spatial localization of brain tumors. Unlike x-ray and CT imaging, MRI provides high-resolution images, with superior soft-tissue contrast without employing ionizing radiation (Banerjee et al., 2020). For diagnosis of brain gliomas, four MRI sequences are routinely acquired, namely, T1-weighted, T1-weighted contrast-enhanced (T1ce), T2 weighted, and Fluid attenuated inversion recovery (FLAIR).

In the management of HGGs, overall survival (OS) plays a critical role in treatment and surgical planning (Puybareau et al., 2018; Sanghani et al., 2018; Feng et al., 2020). OS is usually defined as *the number of days* a patient survives post-surgery (Macyszyn et al., 2015). In the BraTS challenges (2017− 2020)(Bakas et al., 2018; Crimi and Bakas, 2020, 2021), OS prediction in HGGs is formulated as a classification task, and competitors are asked to develop machine learning methodologies as applied to brain MRIs and estimate the OS for each patient. The BraTS challenge defined three survival classes namely, short-term (< 10 months), medium-term (*10-15* months), and long-term (> 15 months) survivors (Bakas et al., 2018).

In the last few years, several machine learning approaches have appeared that predict OS in brain gliomas by directly utilizing information from MRI scans. Among these machine learning methods, the majority are based on radiomic strategies. The publicly available BraTS dataset (Menze et al., 2014; Bakas et al., 2017a, b, c, 2018) that becomes a status quo for brain tumor segmentation and OS classification eases rigorous comparisons of available methods and leads to substantial advances in this field.

### Brief Literature on BraTS

Puybareau et al. (2018) used a 2D fully convolutional neural network (FCNN), based on VGG-16 architecture, for segmentation of brain tumors into three non-overlapping subregions including peritumoral edema (PTE), non-enhancing core (NEC), and enhancing core (ENC). Ten volumetric features were extracted from subjects, with Gross Tumor Resection (GTR) status, using ground truth segmentation maps on the training cohort and the obtained multi-regional segmentation maps on the validation and challenge cohorts. The extracted features were normalized with principal component analysis and used to train *50* random forest classifiers. The final prediction (of survival class) was obtained by a majority vote on the *50* predictions from trained classifiers. The authors reported an accuracy of 37.9% on the validation cohort and 61% on the challenge cohort.

Feng et al. (2020) used an ensemble of six 3D U-Net architectures for the segmentation of brain tumors into three non-overlapping subregions (PTE, NEC, and ENC). A linear regression model was trained with six volumetric features, extracted using multi-regional segmentation maps and clinical features. The study reported an accuracy of 32.1% in the validation cohort and 61% in the challenge cohort. Pei et al. (2020) proposed a 3D self-ensemble ResU-Net architecture for the segmentation of brain tumors into three non-overlapping subregions (PTE, NEC, and ENC). A total of 34 shape-features were extracted from the obtained multi-regional segmentation maps and ranked based on the feature importance attribute of a random forest classifier. The most predictive features were used to train a random forest regressor. The authors reported an accuracy of 55.2% on the validation cohort and 43% on the challenge cohort.

Kao et al. (2018) utilized an ensemble of 26 neural network architectures (19 variants of Deep-Medic (Kamnitsas et al., 2017) and seven variants of 3D U-Net (Çiçek et al., 2016) with random initialization, data augmentation, normalization, and loss function) for segmentation of brain tumor into three non-overlapping subregions (PTE, NEC, and ENC). From the obtained multi-regional segmentation maps, they extracted *19* morphological, *19* volumetric, *78* volumetric spatial, and 116tractography features from *59* subjects with gross tumor resection (GTR) status. Discriminatory features were selected by recursive feature elimination and used to train an SVM classifier with a linear kernel. Compared to morphological, spatial, and volumetric features, tractography features achieved a high accuracy of 69.7% in the training cohort but a low accuracy of 35.7% in the validation cohort and 41.6% in the challenge cohort. Islam et al. (2018) employed PixelNet (Islam and Ren, 2017) for segmentation of brain tumors into three non-overlapping subregions followed by extraction of radiomic features including shape, volumetric, and first order features. A subset of *50* most predictive features was selected using cross-validation and used to train an artificial neural network for prediction. The authors reported an accuracy of 46.8% on the challenge cohort.

McKinley et al. (2020) utilized a 3D-to-2D FCNN for the segmentation of brain tumor into three overlapping subregions, i.e., whole tumor (WT), tumor core (TC), and active tumor (EC). Three features – number of distinct tumor components, number of tumor cores, and age – were used to train a fusion of linear regression and random forest classifiers. The study reported an accuracy of 61.7% on the challenge cohort. Bommineni (2020) used an ensemble of four 3D U-Nets, called Piece-Net, for the segmentation of brain tumors into three non-overlapping subregions. Radiomic and clinical features including volume, surface area, spatial location, and age were used to train a linear regression model. The study reported an accuracy of 37.9% in the validation cohort and 58.9% in the challenge cohort.

Marti Asenjo and Martinez-Larraz Solís (2020) used an ensemble of four U-Net networks (three 2D U-Nets and one 3D U-Net) for the segmentation of brain tumors into three non-overlapping regions. The obtained multi-regional segmentation maps were used to extract a diverse set of radiomic features including first-order, shape, texture, and spatial features. Three models were independently learned for the OS classification task: (1) the RUSboosted decision tree classifier was trained using a subset of *24* predictive features obtained with a chi-square test, (2) the SVM classifier with a quadratic kernel was trained using a subset of *10* predictive features obtained with MRMR method, and (3) regression tree was trained using a subset of *29* predictive features obtained with *F*-test. Discrete label predictions were replaced with (continuous) survival days as follows: *150* days for short-term survivors, *376* days for medium-term survivors, and *796* days for long-term survivors. The final prediction was obtained by taking a mean of the continuous values (survival days) of the three trained models. The study reported an accuracy of 61.7% on the challenge cohort.

### Is Deep Learning Falling Short for Overall Survival Prediction?

Although deep learning methods have achieved state-of-the-art results in numerous applications in clinical and translational imaging, their efficacy in the OS classification task of brain gliomas is yet to be established. Numerous studies have shown that, in comparison to classification models trained with handcrafted (radiomic) features, deep models reported poor predictive performance on BraTS validation and challenge cohorts (Suter et al., 2018; Guo et al., 2019; Starke et al., 2019; Akbar et al., 2020). For instance, Akbar et al. (2020) extracted deep features from 2D multi-parametric MRI scans by employing the modified versions of MobileNet V1 (Howard et al., 2017) and MobileNet V2 (Sandler et al., 2018) architectures. Deep features, augmented with a clinical feature (Age in years), were subsequently fed to a deep learning prediction module called the survival prediction model (SPM). The study reported an accuracy of 31% in the validation cohort and 40.2% in the challenge cohort. Recently, two studies demonstrated the strong performance of deep models for the OS classification task: Zhao et al. (2020) used a deep learning framework, called Segmentation then Prediction (STP), based on 3D U-Net. The STP framework is composed of a segmentation module, which segments the brain tumor volume into overlapping subregions (i.e., WT, TC, EC), a local branch, which extracts features from the whole tumor only, and a global branch which extracts features from the last layer of the segmentation module. Features from global and local branches are fused together to generate survival predictions. The study reported an accuracy of 65.5% in the validation cohort and 44.9% in the challenge cohort. Carmo et al. (2020) employed a 3D U-Net with self-attention blocks for segmentation of brain tumor volume into overlapping subregions followed by prediction of OS class. The study reported an accuracy of 55.2% in the validation cohort and 46.7% in the challenge cohort. It is important to note that the generalizability of deep models varied significantly between validation and challenge cohorts.

Suter et al. (2020) attributed the unsatisfactory performance of deep models, on OS classification tasks, to the poor robustness of deep features. In their extensive empirical study, they studied the robustness of various feature categories using *125* perturbations including varying image resolution, *k*-space subsampling, additive noise, bin width for gray values etc. The study showed that, for the OS classification task, shape features are most robust, with Intraclass Correlation Coefficient ICC ∈ [0.97, 0.99], followed by first order features, with ICC ∈ [0.48, 0.92], texture features such as GLSZM with ICC ∈ [0.28, 0.83], GLCM with ICC ∈ [0.32, 0.82], GLRLM with ICC ∈ [0.30, 0.80], GLDM with ICC ∈ [0.31, 0.78], and deep features with ICC ∈ [0.48, 0.86]. Radiomic signature learned with shape features only have already demonstrated strong performance in the OS classification task (Pérez-Beteta et al., 2017; Puybareau et al., 2018; Agravat and Raval, 2020; Bommineni, 2020; Feng et al., 2020; Pei et al., 2020; Parmar, 2021).

### Summary of Our Contributions

In this study, we used the BraTS *2020* dataset (Menze et al., 2014; Bakas et al., 2017a,b, c, 2018) to reach the following goals:

• Quantitatively evaluate the impact of five state-of-the-art deep segmentation networks on radiomics-based prediction of OS in HGGs.
• Explore the efficacy of the *6-subregions* and *21-subregion*s radiomic models, in the OS classification task, obtained using an anatomy-guided multi-regional segmentation of brain tumor volume.
• Quantitatively evaluate the efficacy of multi-region segmentation maps, obtained with the STAPLE-fusion method, on radiomics-based prediction of OS in HGGs.
• Provide a failure analysis of multi-regional radiomic models for the OS classification task.

To the best of our knowledge, this is the first study that extensively studied the influence of segmentation methods on radiomics-guided prediction of OS in brain gliomas.

## Materials and Methods

### Dataset and Preprocessing

In this study, we made use of the publicly available BraTS *2020* dataset (Menze et al., 2014; Bakas et al., 2017a, b, c, 2018). The training cohort consists of *369* subjects with preoperative 3D multiparametric MRI scans (including T1, T2, T1ce, and FLAIR sequences). Manual segmentation of tumor subregions (including peritumoral edema, non-enhancing core, and enhancing core) was provided and confirmed by expert neuroradiologists (Menze et al., 2014). Out of *369* subjects, *76* are low-grade gliomas (LGGs) and *293* are HGGs. Out of *293* HGGs, complete survival information was provided for *236* subjects and GTR was provided only for *118* subjects. Of the *118* subjects, *42* are short-term survivors, *30* are medium-term survivors, and *46* are long-term survivors.

The validation cohort consists of *125* subjects and GTR status is provided for *29* subjects only. Unlike the training cohort, the validation cohort only contained preoperative 3D multiparametric MRI scans (including T1, T2, T1ce, and FLAIR sequences) and did not include manual segmentation of tumor subregions or survival information. Predictions on the validation cohort can only be evaluated online on the CBICA portal^1^. The challenge cohort consists of *166* subjects and is not publicly available for experiments and evaluation.

The BraTS *2020* dataset also includes subjects from The Cancer Imaging Archive (TCIA) (Clark et al., 2013; Scarpace et al., 2016) and provides a name mapping file that matches the BraTS *2020* subject IDs with the TCIA subject IDs. With the help of matched TCIA subject IDs, we managed to extract survival information and clinical variables of an additional *31* HGGs from the validation cohort of the BraTS *2020* dataset. Of the *31* subjects, *16* are short-term survivors, *3* are medium-term survivors, and *12* are long-term survivors.

To summarize, we used the following data cohorts in our experiments:

(1) Training cohort comprising *118* subjects,
(2) Testing cohort A comprising *31* subjects, and
(3) Testing cohort B comprising *29* subjects.

Manual segmentation of tumor subregions is only available for the training cohort. Survival information is only available for the training cohort and testing cohort A. Table 1 summarizes the demographic and clinical characteristics of the training and testing cohorts.

**TABLE 1:** Summary of training and testing cohorts (A and B) used in the overall survival classification task.

Characteristics	Training cohort	Testing cohort A	Testing cohort B
**Patient demographic**			
No. of patients	*118*	*31*	*29*
Patient distribution			
CBICA UPenn	*94*	–	*15*
TCIA	*17*	*31*	–
Others	*7*	–	*14*
**Imaging data**			
3D multiparametric MRI scans (T1, T1ce, T2, and FLAIR)	√	√	√
Ground truth Segmentation masks	√	×	×
**Clinical information**			
**Age (years) (*p* = 0.252)^1^ (*p* = 0.115)^2^**			
Range	27.8−86.6	17.0−80.0	21.7−85.6
Mean	61.9	58.4	57.3
Median	63.5	*58*	*58*
1-Standard deviation	12.0	15.5	14.3
**Survival groups (*p* = 0.40)^3^**			
Range (days)	*12-1767*	*16-1215*	–
Mean (days)	446.4	390.8	–
Median (days)	374.5	293.7	–
1-Standard deviation (days)	343.8	314.4	–
Short-term [10months]	*42*	*16*	–
Medium-term [10−15months]	*30*	*3*	–
Long-term [ > 15months]	*46*	*12*	–

*^1^p-value for statistical comparison of age between training cohort and testing cohort A.*

*^2^p-value for statistical comparison of age between training cohort and testing cohort B.*

*^3^p-value for statistical comparison of survival between training cohort and testing cohort A.*

The 3D MRI scans for each subject were already skull-stripped, registered to the SRI24 template, and resampled to an isotropic 1mm×1mm×1 mm resolution (Bakas et al., 2017c). The 3D MRI T1 scan for each subject was preprocessed using the N4ITK bias field correction algorithm (Tustison et al., 2010), which is a recommended pre-processing step before performing any medical image processing task such as image registration (Moradmand et al., 2020)^2^.

### Convolutional Neural Networks-Based Segmentation of Brain Tumor Volume

Segmentation of brain tumor volume in 3D multi-parametric MRI scans is the penultimate step in radiomics of brain gliomas. Brain tumor volume can be partitioned into three non-overlapping or three overlapping subregions. The three non-overlapping subregions of brain tumor volume are peritumoral edema (PTE), non-enhancing core (NEC), and enhancing core (ENC). These non-overlapping subregions are combined in various ways to generate three overlapping subregions of brain tumor volume. The whole tumor (WT) is a combination of PTE, NEC, and ENC subregions. The tumor core (TC) is a combination of NEC and ENC. The active tumor (EC) only contains the enhancing core (ENC).

Manual segmentation of tumor subregions are already provided for the training cohort by BraTS challenge organizers. For testing cohorts (A and B), segmentation of brain tumor volume is automatically generated using convolutional neural networks (CNNs) trained on the BraTS 2020 training data (*369* subjects). To study segmentation-induced variability in radiomics performance, we employed five state-of-the-art CNNs for brain tumor segmentation, namely, Dong 2D U-Net (Dong et al., 2017), Wang 2.5D CNN (Wang F. et al., 2019), Isensee 3D U-Net (Isensee et al., 2021), HDC-Net (Luo et al., 2020), and E_1_D_3_ 3D U-Net (Talha Bukhari and Mohy-ud-Din, 2021).

Dong 2D U-Net has shown superior performance on the BraTS 2015 dataset (*274* subjects). Wang 2.5D CNN and HDC Net have shown superior performance on the BraTS 2017 (*487* subjects) and BraTS 2018 (*542* subjects) datasets. Isensee 3D U-Net has shown superior performance on the BraTS 2018 dataset. E_1_D_3_ 3D U-Net has shown superior performance on BraTS 2018 and BraTS 2021 (*2040* subjects) datasets. E_1_D_3_ 3D U-Net, Isensee 3D U-Net, and Wang 2.5D CNN yield overlapping subregions (WT, TC, and EC), and Dong 2D U-Net and HDC-Net provide non-overlapping subregions (PTE, NEC, and ENC) of brain tumor volume. It must be noted that one can easily obtain non-overlapping subregions from overlapping ones *via* set-subtraction.

Dong et al. (2017) proposed a slice-based 2D U-Net to segment tumor sub-regions on volumetric MRI scans. Only two MRI sequences were used, namely FLAIR and T1ce. The FLAIR sequence was used to delineate WT and TC subregions and the T1ce sequence was used to segment the EC subregion. An extensive data augmentation pipeline was employed to improve the generalization performance of 2D U-Net. Wang F. et al. (2019) developed a hierarchical region-based tumor segmentation approach by using a cascade of 2.5D CNN networks. Each CNN used anisotropic pseudo-3D convolution kernels with multi-scale prediction and used multi-view fusion (along axial, coronal, and sagittal directions) to generate segmentation of a tumor sub-region. Three such segmentation frameworks, namely W-Net, T-Net, and E-Net, were combined in a cascade where one works on the output of the previous one to yield a multi-class segmentation map. Isensee et al. (2021) used a meticulously tuned 3D U-Net architecture for brain tumor segmentation. In addition to empirically tuned hyperparameters, it included instance normalization, region-based prediction, data augmentation, and post-processing. Isensee 3D U-Net yields a non-overlapping segmentation of brain tumor subregions (PTE, NEC, ENC). Luo et al. (2020) proposed a hierarchically decoupled CNN (HDC-Net) by replacing standard convolution blocks in a 3D U-Net with new lightweight HDC blocks composed of carefully arranged 2D convolutions. HDC blocks have a small parameter count and work simultaneously for the channel and spatial dimensions. Talha Bukhari and Mohy-ud-Din (2021) proposed a modification of the 3D U-Net inspired by the concept of TreeNets and Region-based prediction. The proposal, named E_1_D_3_, is a single encoder multi-decoder architecture, where each decoder segments one of the three hierarchical tumor sub-regions: WT, TC, and EC. The three binary segmentation maps are then fused together through a combination of morphological processing, cluster thresholding, and hierarchy imposing operations to generate a multi-class segmentation map.

We also employed the STAPLE-fusion method (Rohlfing et al., 2004) to fuse the segmentation labels obtained from Dong 2D U-Net, Wang 2.5D CNN, Isensee 3D U-Net, HDC-Net, and E_1_D_3_ 3D U-Net. Overall, we used six segmentation schemes, five CNNs, and one STAPLE-fusion method. Configuration and hyperparameters for the five segmentation architectures are presented in Table 2.

**TABLE 2:** Configuration and hyperparameters of five CNNs used for automatic segmentation of brain tumor volume.

Network	Dong 2D U-Net	Wang 2.5D CNN	Isensee 3D U-Net	HDC-Net	E_1_D_3_ 3D U-Net
Architecture	2D U-Net	Three 2.5D Anisotropic CNNs (W-Net, T-Net, and E-Net) in cascade	3D U-Net with Deep supervision	2.5D U-Net	3D U-Net
Activation	ReLU	P-ReLU	Leaky-ReLU (0.01)	ReLU	Leaky-ReLU (0.01)
Batch size	10	5 (Same for three CNNs in cascade)	2	8	2
Initialization	He-normal	Truncated Normal	He-normal	He-normal	He-normal
Input size/Output size	240^2^/240^2^	W-Net: 19×144^2^ /11×144^2^ T-Net: 19×64^2^ /11×64^2^ E-Net: 19×64^2^ /11×64^2^	128^3^/128^3^	128^3^/128^3^	96^3^/96^3^
Learning Rate policy^1^	Polynomial Decay (batch-wise) η_0_ = 10^−4^η_*end*_ = 10^−7^γ = 1.2	Constant (10^−3^)	Polynomial decay (epoch-wise) η_0_ = 0.01γ = 0.9	Polynomial decay (epoch-wise) η_0_ = 10^−3^γ = 0.9	Polynomial decay (epoch-wise) η_0_ = 10^−2^γ = 0.9
Optimizer	Adam	Adam	SGD + Nesterov (0.99)	Adam (AMSGrad variant)	SGD + Nesterov (0.99)
Loss	Soft Dice	Soft Dice	Soft Dice + Cross Entropy	Generalized Soft Dice	Soft Dice + Cross Entropy
Regularization	–	*L*_2_ (10^−7^)	*L*_2_ (3×10^−5^)	*L*_2_ (10^−5^)	*L*_2_(10^−6^)
Total Training iterations (Gradient-Decent updates)	50k (100 epochs)	20k (per network)	250k (1000 epochs)	37.35k (900 epochs)	125k (500 epochs)
# Parameters	34.5*million*	W-Net: 0.21*million* T-Net: 0.21*million* E-Net: 0.20*million*	31.2*million*	0.29*million*	34.9million
Training Time^2^	∼110 h	W-Net (single-view): ∼84 h T-Net (single-view): ∼84 h E-Net (single-view): ∼20 h	∼101 h	∼110 h	∼48 h
Test-time Augmentation	√	×	√	√	√
Morphological Post-processing	Morphological closing, cluster thresholding	×	×	×	√

*^1^For definition of variables consult Table 1 in Bukhari and Mohy-ud-Din (2021).*

*^2^The training time also depends on the GPU system used for training. HDC-Net was trained on a dual-GPU system, whereas the remaining CNNs were trained on a single-GPU system.*

### Tumor Subregion Segmentation Models

We focused on two models for the segmentation of tumor subregions in brain gliomas: (1) physiology-based segmentation and (2) anatomy-based segmentation.

#### Physiology-Based Segmentation Model

The brain tumor is divided into three non-overlapping subregions (PTE, NEC, and ENC). We hypothesized that features from tumor subregions are localized as compared to the whole tumor region. Tumor subregions can potentially provide better features that are consistent with the prognosis of the tumor (Macyszyn et al., 2015; Li, 2018). In this study, the physiology-guided overlapping segmentation model is referred to as the *3-subregions* model.

#### Anatomy-Based Segmentation Models

The brain tumor is subdivided into anatomical regions with the help of a pre-defined Harvard-Oxford subcortical atlas with 21 labeled anatomical regions (Desikan et al., 2006). Anatomy-based segmentation is obtained in four steps: (1) Harvard-Oxford subcortical atlas is registered into subject space using diffeomorphic registration. To do this, we used the SyNOnly algorithm as implemented in the ANTs (Advanced Normalization Tools) package (Avants et al., 2009). SyNOnly was initialized with the output of affine registration and used mutual information as a cost function. (2) The whole tumor (WT) mask is overlaid with the registered atlas to extract the tumor-affected anatomical regions. (3) Volumes of tumor-affected anatomical regions are computed and then ranked in descending order. (4) Finally, the top-*K* anatomical subregions that combine to occupy more than *85*% of WT volume are retained. In this study, we refer to this segmentation scheme as *6-subregions* segmentation model where 6(= *K*) is the number of subregions selected in step 4. For comparison, we also used the *21-subregions* segmentation model obtained in step 2.

Given a segmentation model, i.e., *3-subregions*, *6-subregions, or 21-subregions*, one can extract region-specific radiomic features for classification.

### Radiomic Feature Extraction

To compare the power of region-specific radiomic models, we extracted radiomic features, using the *PyRadiomics* software package (Van Griethuysen et al., 2017), from WT only (referred to as the *WT radiomics model*), from three non-overlapping subregions, i.e., PTE, NEC, and ENC (referred to as the *3-subregions radiomics model*), from six anatomical subregions, i.e., left and right cerebral cortex, left and right cerebral white matter, and left and right lateral ventricle (referred to as the *6-subregions radiomics model*), and from 21 anatomical subregions provided by the registered Harvard-Oxford subcortical atlas (referred to as the *21-subregions radiomics model*). We extracted the following set of (radiomic) features:

#### Shape Features

Shape features include volume and surface area of each subregion. For instance, in a *3-subregions* radiomics model, we extract the volume and surface area of the peritumoral edema, the non-enhancing core, and the enhancing core. In the OS classification task, shape features have been shown to provide insights into tumor behavior (Puybareau et al., 2018; Agravat and Raval, 2020; Bommineni, 2020; Pei et al., 2020; Parmar, 2021). Several studies reported that tumor volume and surface area are strong predictors of survival in patients with glioblastoma (Menze et al., 2014; Wang G. et al., 2019; Bommineni, 2020; Feng et al., 2020). A large tumor volume reflects the severity of the tumor and is associated with poor prognosis and shorter survival times (Menze et al., 2014; Feng et al., 2020). We extracted *2* shape features for the WT radiomics model, *6* shape features for the *3-subregions* radiomics model, *12* for the *6-subregions* radiomics model, and *42* shape features for the *21-subregions* radiomics model.

#### Spatial Features

Spatial features capture the location of the tumor within the brain. More specifically, we extract (1) coordinates (in 3D) of the centroid of the WT with respect to the brain mask and (2) the Euclidean distance between the centroid of the WT and the centroid of the brain mask. A brain mask is defined as the non-zero region in the 3D FLAIR sequence. Spatial features have been shown to be predictive for survival prediction tasks (Carver et al., 2018; Puybareau et al., 2018; Bommineni, 2020).

#### Demographic Feature

We include age (in years), which is provided for each subject in the BraTS 2020 dataset.

Combined with the spatial and clinical features, a total of 7 features were obtained for the *WT* radiomics model, *11* features for the *3-subregions* radiomics model, *17* for the *6-subregions* radiomics model, and 47 features for the *21-subregions* radiomics model. A summary of the radiomic features is provided in Table 3.

**TABLE 3:** Summary of radiomic features extracted for four radiomic models, namely, the *WT* radiomics model, the *3-subregions* radiomics model, the *6-subregions* radiomics model, and the *21-subregions* radiomics model.

Feature types	Feature names	No of features
Clinical features	Age	1
Spatial features	Centroid of the WT, (Euclidean) Distance between the (centroid of) WT and the (centroid of) the brain	4
Shape features (WT radiomics model)	Volume and Surface Area of Whole Tumor	2
Shape features (*3-subregions* radiomics model)	Volume and Surface Area of Peritumoral Edema (PTE), Enhancing Core (ENC), and Non-Enhancing Core (NEC)	6 (2 features × 3 subregions)
Shape features (*6-subregions* radiomics model)	Volume and Surface Area of Right Cerebral Cortex (RCC), Left Cerebral Cortex (LCC), Left Lateral Ventricle (LLV), Right Lateral Ventricle (RLV), Left Cerebral White Matter (LCWM), Right Cerebral White Matter (RCWM)	12 (2 features × 6 subregions)
Shape features (*21-subregions* radiomics model)	Volume and Surface Area of 21 Subcortical Regions defined by a registered Harvard-Oxford subcortical atlas (see Appendix)	42 (2 features × 21 subregions)

For the training cohort, shape and spatial features were extracted from manual segmentations provided with the BraTS 2020 dataset. For testing cohorts (A and B), shape and spatial features were extracted from predicted segmentations obtained using six segmentation schemes (five CNNs and one STAPLE-fused segmentation) elaborated in Section “CNN-based Segmentation of Brain Tumor Volume.”

### Training and Inference

Every feature vector, from the training cohort, was independently normalized (i.e., transformed to *z*-scores) by subtracting the mean and dividing by the standard deviation. Features from testing cohorts A and B were normalized using the mean and standard deviation of the training cohort. For the radiomic models (i.e., *WT* radiomics model, *3-subregions* radiomics model, *6-subregions* radiomics model, and *21-subregions* radiomics model), no feature selection was performed.

For the training phase, random forest classifiers (*N* = 100) were trained on the training cohort comprising *118* subjects with GTR status. No synthetic data oversampling was performed because the training cohort was well-balanced across the three survival classes (Shannon’s entropy = 0.97). Hyperparameters of each random forest classifier were set as follows: no_of_estimators = 200, max_features = auto, class_weight = balanced, and criterion = gini. For the inference phase, a soft voting method was adopted to unify the outputs of *N* random forest classifiers (with a uniform weighting scheme) and generate a single prediction of OS class for each subject. To monitor the overfitting of the radiomic models in the inference phase, we also evaluated predictive performance with *200* times repeated stratified splitting (70−30%) of the training cohort (*118* subjects).

### Evaluation Criteria

The performance of the six segmentation schemes (five CNNs and one STAPLE-fusion method) was quantified using Dice Similarity Coefficient (DSC) (Dice, 1945) and Hausdorff distance metric (HD-95) (Huttenlocher et al., 1993). The six segmentation schemes were ranked based on the Final Ranking Score (FRS), and statistical significance (of ranking) was calculated using a random permutation test (Bukhari and Mohy-ud-Din, 2021). In testing cohort A (*31* subjects), the predictive performance of radiomic models was quantified using the area under the receiver operating curve (AUC) and the area under the precision-recall curve (AUPRC). In testing cohort B (*29* subjects), the predictive performance of radiomic models could only be quantified with the accuracy metric on the CBICA online portal. The stability of the radiomic models was quantified with relative standard deviation (RSD) calculated as a ratio of standard deviation to the mean of AUC. A lower value of RSD corresponds to the higher stability of the radiomic models. Statistical analysis of demographic data (in Table 1) was performed using the student *t*-test. A *p*−*value* < 0.05 was considered statistically significant and a *p*−*value* < 0.001 was considered statistically highly significant.

### System Specifications

All experiments were implemented in Python 3.6 using the following open-source packages: scikit-learn (Pedregosa et al., 2011), N4ITK bias field correction (Tustison et al., 2010), ANTs (Avants et al., 2009), PyRadiomics^3^ (Van Griethuysen et al., 2017), Pandas (McKinney, 2010), Nibabel^4^, and STAPLE-fusion^5^ (Rohlfing et al., 2004).

## Results

### Clinical Characteristics

Table 1 displays the clinical characteristics of the training cohort and testing cohorts. The median age of the training cohort, testing cohort A, and testing cohort B were 63.5, *58*, and *58* years, respectively. No statistical difference was found in age between the training cohort and the testing cohort A (*p* = 0.252) and the training cohort and the testing cohort B (*p* = 0.115). The median OS (in days) for the training cohort and the testing cohort A were *375* days and *294* days, respectively. While the training cohort was balanced across three survival groups, i.e., short-term (*42* subjects), medium-term (*30* subjects), and long-term (*46* subjects) survivors, testing cohort A had a sparse presence of medium-term survivors – only *3* subjects out of *31*. No statistical difference was found in OS days between the training cohort and testing cohort A (*p* = 0.40). Survival information was not made publicly available for testing cohort B by the BraTS 2020 organizers.

### Brain Tumor Segmentation

The performance of the six segmentation schemes (five CNNs and one STAPLE-fusion method), for testing cohorts A and B combined (*60* subjects), is summarized in Table 4. We used Final Ranking Score (FRS) to unify the six segmentation performance metrics (i.e., DSC and HD-95 scores for three subregions each) for each subject in testing cohorts A and B (Bukhari and Mohy-ud-Din, 2021).

**TABLE 4:** Performance of six segmentation schemes, including five CNNs and one STAPLE-fusion method, on testing cohorts A and B (60 subjects).

Segmentation network	Dice similarity coefficient (%)			Hausdorff distance (mm)			Final Ranking Score (FRS)
		
	WT	TC	EC	WT	TC	EC	
Dong 2D U-Net	90.4 ± 6.5	87.3 ± 9.9	84.1 ± 9.4	5.8 ± 9.0	6.5 ± 8.6	3.2 ± 5.5	6**
Wang 2.5D CNN	90.6 ± 5.5	89.3 ± 8.7	85.2 ± 10.0	6.6 ± 10.0	5.7 ± 8.7	2.9 ± 4.8	5**
Isensee 3D U-Net	**91.5** ± **5.5**	**90.9** ± **6.7**	**87.0** ± **7.3**	4.4 ± 5.6	4.4 ± 8.5	2.1 ± 1.9	1
HDC-Net	90.8 ± 5.4	90.1 ± 7.3	85.9 ± 8.4	4.3 ± 4.4	4.5 ± 8.0	2.1 ± 1.3	3**
E_1_D_3_ 3D U-Net	91.4 ± 4.9	89.7 ± 9.0	85.9 ± 9.1	5.5 ± 7.8	5.6 ± 10.0	3.4 ± 6.3	4**
STAPLE-Fusion	91.4 ± 4.8	90.6 ± 7.6	86.7;±7.7	**4.1** ± **3.4**	**4.4** ± **8.1**	**2.0** ± **1.3**	2

*Bold font indicates best scores for overlapping subregions (WT, TC, and EC).*

**Indicates that the segmentation network is ranked significantly lower (p < 0.001) in comparison to the top-ranked method Isensee 3D U-Net (FRS = 1).

In terms of FRS, Isensee 3D U-Net was ranked significantly higher (*p* < 0.001) in comparison to the remaining CNNs for brain tumor segmentation. Isensee 3D U-Net obtained the highest DSC scores for WT (DSC =91.5), TC (DSC =90.9), and EC (DSC =87.0) subregions which quantify overlap with manual segmentation maps. In terms of the HD-95 metric, Isensee 3D U-Net was quite close in performance to HDC-Net (ΔHD_avg_ = 0.07) and much better than E_1_D_3_ 3D U-Net (ΔHD_avg_ = 1.2), Wang 2.5D CNN (ΔHD_avg_ = 1.43), and Dong 2D U-Net (ΔHD_avg_ = 1.53).

**FIGURE 1:**
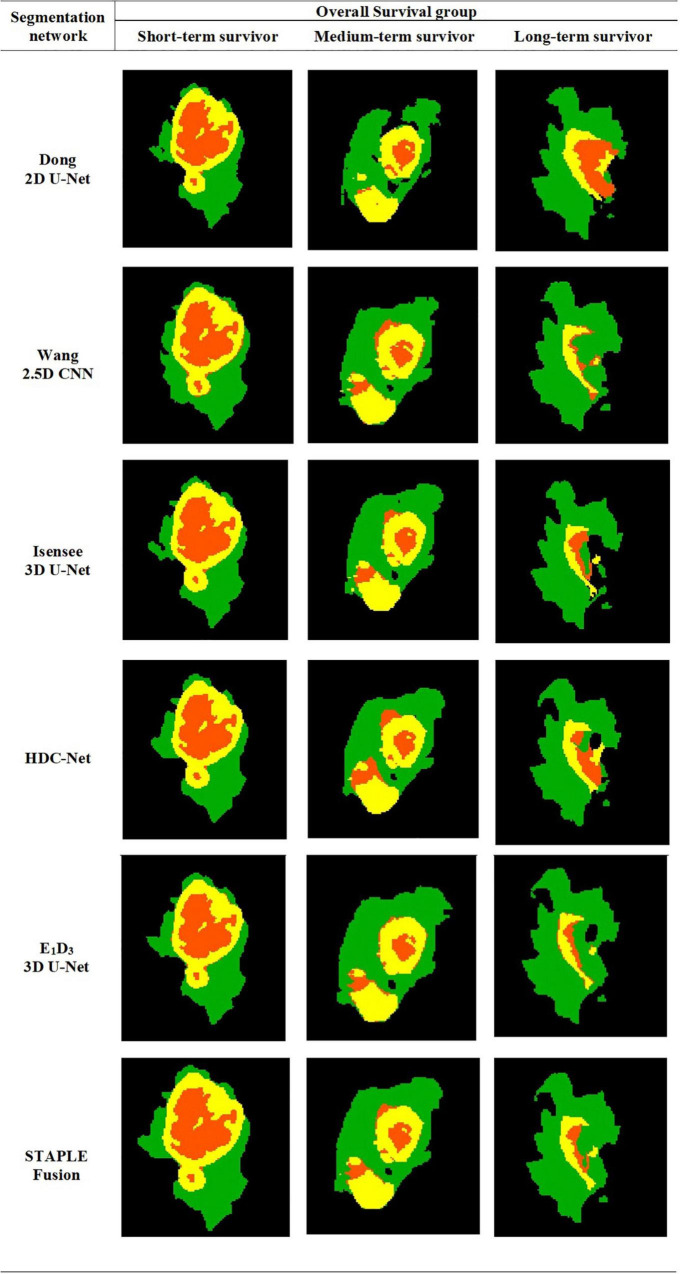
Automatically segmented tumor subregions from six segmentation schemes including five CNNs and one STAPLE fusion method. Peritumoral edema, enhancing core, non-enhancing core. peritumoral edema (Green) enhancing core (Yellow) and non-enhancing core (Orange).

The STAPLE-fusion method ranked second, in terms of FRS, but not significantly lower than Isensee 3D U-Net (*p* = 0.205). However, the STAPLE-fusion method was ranked significantly higher than Dong 2D U-Net (*p* < 0.001), Wang 2.5D CNN (*p* < 0.001), HDC-Net (*p* < 0.001), and E_1_D_3_ 3D U-Net (*p* < 0.001). Compared to the five CNNs (individually), the STAPLE-fusion method reported the lowest HD-95 scores which measure the degree of mismatch between manual and predicted segmentation maps. Figure 1 shows the predicted multi-class segmentation maps obtained with six segmentation schemes for three subjects, one from each survival class, in testing cohort A.

### Evaluation on Training Cohort

The four radiomic models, i.e., the *WT* radiomics model, the *3-subregions* radiomics model, the *6-subregions* radiomics model, and the *21-subregions* radiomics model, reported the following predictive performance in the training cohort: (1) The *WT* radiomics model reported an AUC = 0.63 ± 0.06 and AUPRC = 0.49 ± 0.07, (2) The *3-subregions* radiomics model reported an AUC = 0.68 ± 0.05 and AUPRC = 0.53 ± 0.07, (3) The *6-subregions* radiomics model reported an AUC = 0.61 ± 0.06 and AUPRC = 0.45 ± 0.06, and (4) The *21-subregions* radiomics model reported an AUC = 0.58 ± 0.05 and AUPRC = 0.42 ± 0.06. The predictive performance of each radiomics model was averaged over *200* times by repeated stratified splitting of the training cohort (70−30%).

### Evaluation on Testing Cohort A (31 Subjects)

Performance measures (AUC, AUPRC, and RSD) for the four radiomic models, i.e., *WT* radiomics model, *3-subregions* radiomics model, *6-subregions* radiomics models, and *21-subregions* radiomics model, are summarized in Table 5.

**TABLE 5:** Quantitative analysis of four radiomic models, namely, the *WT* radiomics model, the *3-subregions* radiomics model, the *6-subregions* radiomics model, and the *21-subregions* radiomics model on testing cohort A (31 subjects).

Segmentation network	Performance metric	WT radiomics model	*3-Subregions* radiomics model	*6-Subregions* radiomics model	*21-Subregions* radiomics model
Dong 2D U-Net	AUC	**0.70**(0.71, 0.66, 0.69)	**0.75** (0.75, 0.46, 0.77)	0.71(0.77, 0.48, 0.70)	0.70(0.68, 0.43, 0.78)
	AUPRC	0.58	0.66	0.51	0.57
Wang 2.5D CNN	AUC	0.68(0.68, 0.44, 0.7)	**0.75** (0.66, 0.48, 0.87)	0.70(0.71, 0.42, 0.74)	0.70(0.65, 0.43, 0.78)
	AUPRC	0.53	0.68	0.51	0.56
Isensee 3D U-Net	AUC	**0.70** (0.72, 0.38, 0.72)	0.71(0.7, 0.31, 0.75)	**0.73** (0.75, 0.44, 0.78)	**0.72** (0.69, 0.45, 0.78)
	AUPRC	0.57	0.62	0.56	0.61
HDC-Net	AUC	0.69(0.68, 0.45, 0.72)	0.73(0.67, 0.53, 0.82)	0.71(0.71, 0.45, 0.75)	0.71(0.67, 0.45, 0.78)
	AUPRC	0.54	0.61	0.53	0.58
E_1_D_3_ 3D U-Net	AUC	0.67(0.68, 0.36,0.69)	0.72(0.71, 0.35, 0.8)	0.71(0.73, 0.42, 0.76)	**0.72** (0.69, 0.51, 0.79)
	AUPRC	0.54	0.64	0.57	0.60
STAPLE Fusion	AUC	0.68(0.69, 0.42, 0.71)	0.74(0.7, 0.45, 0.82)	0.70(0.75, 0.40, 0.74)	0.71(0.69, 0.43, 0.77)
	AUPRC	0.55	0.64	0.51	0.59

*Bold font indicates the best performance achieved for each radiomics model.*

*The micro-AUC of the three classes is displayed as an ordered triplet (short-term survivor, medium-term survivor, and long-term survivor) below the weighted average AUC value.*

*Note: For compactness of description, we refer to the radiomics model, trained with features extracted from the segmentation map generated by a particular segmentation scheme, by the specific name of the segmentation scheme.*

#### Whole Tumor Radiomics Model

Our results showed that Dong 2D U-Net and Isensee 3D U-Net showed the highest predictive performance (AUC = 0.70 and AUPRC = 0.58) and E_1_D_3_ 3D U-Net showed the lowest predictive performance (AUC = 0.67 and AUPRC = 0.54). While Isensee 3D U-Net showed strong predictive power for short-term survivors (AUC = 0.72) and long-term survivors (AUC = 0.72), its performance dropped considerably for medium-term survivors (AUC = 0.38). Dong 2D U-Net displayed the best predictive performance for medium-term survivors (AUC = 0.66) while maintaining high predictive performance on short-term survivors (AUC = 0.71) and long-term survivors (AUC = 0.69). The stability of the *WT* radiomics model was 1.52 as measured with RSD, across the six segmentation methods. The STAPLE-fusion method marginally exceeded the predictive performance of E_1_D_3_ 3D U-Net and was inferior to the remaining segmentation schemes.

#### *3-Subregions* Radiomics Model

Our results showed that Wang 2.5D CNN and Dong 2D U-Net showed the highest predictive performance (AUC = 0.75 and AUPRC = 0.68) and Isensee 3D U-Net showed the lowest predictive performance (AUC = 0.71 and AUPRC = 0.62). While Dong 2D U-Net showed strong predictive power for short-term survivors (AUC = 0.75) and long-term survivors (AUC = 0.77), its performance dropped considerably for medium-term survivors (AUC = 0.46). HDC-Net displayed the best predictive performance for medium-term survivors (AUC = 0.53) while maintaining high predictive performance on long-term survivors (AUC = 0.82) and short-term survivors (AUC = 0.67). The stability of the *3-subregions* radiomics model was 1.99 as measured with RSD, across the six segmentation methods. The STAPLE-fusion method exceeded the predictive performance of E_1_D_3_ 3D U-Net, HDC-Net, and Isensee 3D U-Net and was inferior to the remaining segmentation schemes.

#### *6-Subregions* Radiomics Model

Our results showed that Isensee 3D U-Net showed the highest predictive performance (AUC = 0.73 and AUPRC = 0.56) and Wang 2.5D CNN showed the lowest predictive performance (AUC = 0.70 and AUPRC = 0.51). Dong 2D U-Net showed the best predictive performance for medium-term survivors (AUC = 0.48) while maintaining strong performance on short-term survivors (AUC = 0.77) and long-term survivors (AUC = 0.70). The stability of the *6-subregions* radiomics model, across the six segmentation methods, was 1.48. The predictive performance of the STAPLE-fusion method was similar to Wang 2.5D U-Net and inferior to the remaining segmentation schemes.

#### *21-Subregions* Radiomics Model

Our results showed that Isensee 3D U-Net and E_1_D_3_ 3D U-Net showed the highest predictive performance (AUC = 0.72 and AUPRC = 0.61) and Dong 2D U-Net and Wang 2.5D CNN showed the lowest predictive performance (AUC = 0.70 and AUPRC = 0.57). E_1_D_3_ 3D U-Net showed the best predictive performance for medium-term survivors (AUC = 0.51) while maintaining strong performance on short-term survivors (AUC = 0.69) and long-term survivors (AUC = 0.79). The stability of the *21-subregions* radiomics model, across the six segmentation methods, was 1.39. STAPLE-fusion method marginally exceeded the predictive performance of Dong 2D U-Net and Wang 2.5D U-Net and was inferior to the remaining segmentation schemes.

### Failure Analysis

We performed failure analysis by studying subjects that were misclassified by the radiomic models trained on features extracted from the segmentation maps obtained with six segmentation schemes (five CNNs and one STAPLE-fusion method). More specifically, for each radiomics model, we identified subjects misclassified with (a) all six segmentation schemes (*0-6*), (b) five segmentation schemes (*1-5*), and (c) four segmentation schemes (*2-4*).

**FIGURE 2:**
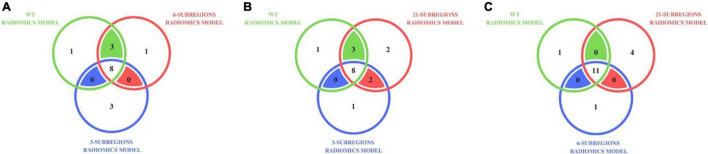
Distribution of misclassified subjects in **(A)** the *WT* radiomics model, the *3-subregions* radiomics model, and the *6-subregions* radiomics model **(B)** the *WT* radiomics model, the *3-subregions* radiomics model, and the *21-subregions* radiomics model **(C)** the *WT* radiomics model, the *6-subregions* radiomics model, and the *21-subregions* radiomics model, on testing cohort A (31 subjects). For instance, 11 subjects were misclassified by the *WT*, *6-subregions*, and *21-subregions* radiomics models.

Our analysis with *WT* radiomics model, the *3-subregions* radiomics model and the *6-subregions* radiomics model revealed that *16* (distinct) subjects were misclassified for at least one radiomics model. Out of *16* subjects, *8* were short-term survivors, *3* were medium-term survivors, and *5* were long-term survivors. Figure 2A shows a Venn diagram that distributes the *16* misclassified subjects across three radiomic models. It also shows that *8* out of *16* subjects were misclassified by all three radiomic models.

Our analysis with the *WT* radiomics model, the *3-subregions* radiomics model, and the *21-subregions* radiomics model revealed that *17* (distinct) subjects were misclassified for at least one radiomics model. Out of *17* subjects, nine were short-term survivors, three were medium-term survivors, and five were long-term survivors. Figure 2B shows a Venn diagram that distributes the *17* misclassified subjects across three radiomic models. It also shows that *8* out of *17* subjects were misclassified by all three radiomic models.

Our analysis with the *6-subregions* radiomics model and the *21-subregions* radiomics model revealed that *16* (distinct) subjects were misclassified for at least one radiomics model. Out of *16* subjects, nine were short-term survivors, two were medium-term survivors, and five were long-term survivors. Figure 2C shows a Venn diagram that distributes the misclassified subjects across the *WT* radiomics model, the *6-subregions* radiomics model, and the *21-subregions* radiomics model. Most subjects (*11* out of *12*) misclassified by the *WT* radiomics model also failed with the *6-subregions* radiomics model and the *21-subregions* radiomics model.

### Evaluation on Testing Cohort B (29 Subjects)

Classification accuracy for the four radiomic models, i.e., *WT* radiomics model, *3-subregions* radiomics model, *6-subregions* radiomics model, and *21-subregions* radiomics model, is summarized in Table 6. Testing cohort B could only be evaluated online on the CBICA portal, which only reported classification accuracy.

**TABLE 6:** Quantitative analysis of four radiomic models, namely, the *WT* radiomics model, the *3-subregions* radiomics model, the *6-subregions* radiomics model, and the *21-subregions* radiomics model, on testing cohort B (29 subjects).

Segmentation network	Accuracy (%)			
	
	WT radiomics model	*3-Subregions* radiomics model	*6-Subregions* radiomics model	*21-Subregions* radiomics model
Dong 2D U-Net	44.8	41.4	48.3	51.7
Wang 2.5D CNN	44.8	41.4	41.4	37.9
Isensee 3D U-Net	44.8	41.4	48.3	44.8
HDC-Net	48.3	37.9	44.8	44.8
E_1_D_3_ 3D U-Net	48.3	44.8	48.3	51.7
STAPLE Fusion	48.3	41.4	48.3‘	41.4

In terms of accuracy, the four radiomic models reported superior (and matched) performance with multiple segmentation schemes. For the *WT* radiomics model, HDC-Net, E_1_D_3_ 3D U-Net, and STAPLE-fusion obtained the highest accuracy (48.3%). For the *3-subregions* radiomics model, the highest accuracy (44.8%) was obtained with E_1_D_3_ 3D U-Net. For the *6-subregions* radiomics model, the highest accuracy of 48.3% was obtained with Dong 2D U-Net, Isensee 3D U-Net, E_1_D_3_ 3D U-Net, and STAPLE-fusion. For the *21-subregions* radiomics model, the highest accuracy of 51.7% was obtained with Dong 2D U-Net and E_1_D_3_ 3D U-Net.

Amongst the six segmentation schemes, E_1_D_3_ 3D U-Net obtained the highest accuracy for the *WT* radiomics model (48.3%), the *3-subregions* radiomics model (44.8%), the *6-subregions* radiomics model (48.3%), and the *21-subregions* radiomics model (51.7%).

## Discussion

In this study, we explored the efficacy of four radiomic models – the *WT* radiomics model, the *3-subregions* radiomics model, the *6-subregions* radiomics model, and the *21-subregions* radiomics model–for OS classification task in brain gliomas. The *WT* radiomics model extracts features from the WT region only. The *3-subregions* radiomics model extracts features from three non-overlapping subregions of WT i.e., PTE, NEC, and ENC. The *6-subregions* radiomics model extracts features from six anatomical regions overlapping with WT volume including left and right cerebral cortex, the left and right cerebral white matter, and the left and right lateral ventricle subregions. The *21-subregions* radiomics model extracts features from 21 anatomical regions provided with the Harvard-Oxford subcortical atlas (see Appendix for the names of 21 anatomical regions). We also quantified the stability of radiomic models across six segmentation schemes–five CNNs and one STAPLE-fusion method. The five CNNs include three 3D CNNs – Isensee 3D U-Net, E_1_D_3_ 3D U-Net, and HDC-Net– one 2.5D CNN, Wang 2.5D CNN, and one 2D CNN, Dong 2D U-Net. For each subject in testing cohorts A and B, the predicted segmentation maps from five CNNs were fused using the STAPLE-fusion method.

We benefitted from the publicly available BraTS 2020 and TCIA datasets and extracted three data cohorts – training cohort (*118* subjects), testing cohort A (*31* subjects), and testing cohort B (*29* subjects). The training cohort comprised of HGGs with 3D multiparametric MRI scans and manual segmentation of brain tumor volume into three non-overlapping subregions, i.e., PTE, NEC, and ENC. Testing cohorts A and B also comprised of HGGs but only included 3D multiparametric MRI scans. While the training cohort was reasonably balanced for the three survival classes – short-term survivors (*42*), medium-term survivors (*30*), and long-term survivors (*46*) – testing cohort A had a sparse representation of medium-term survivors with only *3* subjects out of *31*. OS information for testing cohort B was not available offline.

Segmentation of brain tumor volume is the penultimate step in any radiomics framework for brain gliomas. For each subject in testing cohorts A and B, the brain tumor volume was segmented into three non-overlapping regions (PTE, NEC, and ENC) using the aforementioned six segmentation schemes. Our results showed that 3D CNNs, including Isensee 3D U-Net, HDC-Net, and E_1_D_3_ 3D U-Net, provided superior segmentation of brain tumor subregions by utilizing 3D contextual information in volumetric scans. Among the five CNNs employed for brain tumor segmentation, E_1_D_3_ 3D U-Net had a large memory footprint (*35* million trainable parameters) and the shortest training time (*48* h), and HDC-Net had the fewest trainable parameter (0.29 million trainable parameters) with the long training time (*110* hours). The STAPLE-fusion method significantly outperformed four (of the five) CNNs (*p* < 0.001) except for Isensee 3D U-Net, which was ranked higher (*p* = 0.205). Moreover, the STAPLE-fusion method reported the lowest HD-95 scores, which has been observed previously with ensemble methods (Fidon et al., 2020; Ghaffari et al., 2020; Nguyen et al., 2020; Yang et al., 2020). Isensee 3D U-Net superior performance is attributed to the fact that the underlying 3D U-Net architecture was carefully optimized by empirically tuning network and training parameters on the BraTS dataset.

The *WT* radiomics model, the *6-subregions* radiomics model, and the *21-subregions* radiomics model required accurate segmentation of WT volume which, in terms of Dice score, was performed quite similarly by the six segmentation schemes (DSC: 90.4−91.5%). However, in terms of Hausdorff distance – which measures the largest segmentation error – segmentation of WT volume had a large variability across six segmentation schemes (HD-95: 4.1−6.6mm). The *3-subregions* radiomics model required accurate delineation of additional subregions including tumor core (TC) and active tumor (EC). The segmentation of the TC subregion varied substantially across the six segmentation schemes, in terms of DSC (87.3−90.9%) and HD-95 (4.4−6.5mm) metrics. The segmentation of the EC subregion is increasingly difficult because of poor contrast and fragmented (physiologic) structure. This was exhibited by reduced segmentation accuracy (DSC: 84.1−87.0%) across the six segmentation schemes.

The four radiomic models were obtained by training random forest classifiers (*N=100* for each radiomics model) using shape, volumetric, spatial, and demographic features. Our results showed that the *3-subregions* radiomics model reported superior predictive performance (*meanAUC* = 0.73), across the six segmentation schemes, compared to the *WT* radiomics model (*meanAUC* = 0.69), the *6-subregions* radiomics model (*meanAUC* = 0.71), and the *21-subregions* radiomics model (*meanAUC* = 0.71). This implied that a physiological segmentation of brain tumor volume into three subregions (WT, TC, and EC) played a pivotal role in the OS classification of brain gliomas. The *21-subregions* radiomics model reported the most stable predictions (RSD = 1.39), across six segmentation schemes, compared to the *6-subregions* radiomics model (RSD = 1.48), the *WT* radiomics model (RSD = 1.52), and the *3-subregions* radiomics model (RSD = 1.99). The stability of the *21-subregions* radiomics model and the *6-subregions* radiomics model, over the *3-subregions* radiomics model, is attributed to the sole dependence on the segmentation of WT volume, which is more accurately generated by CNNs compared to TC and EC subregions. It should be noted that physiological segmentation of WT volume (into three non-overlapping subregions) led to more predictive radiomic models and anatomical segmentation of WT volume (into 21 non-overlapping anatomical regions) led to more stable radiomic models.

Our failure analysis with the *WT* radiomics model, the *3-subregions* radiomics model, the *6-subregions* radiomics model, and the *21-subregions* radiomics model revealed that *18* (distinct) subjects were misclassified by at least one radiomic model for a majority of segmentation schemes. We found that the Hausdorff distance metric could be used to explain the aforementioned phenomena. More specifically, we focused on the HD-95 metric for WT segmentation which is common to the three radiomic models. Our analysis showed that the mean HD-95 metric (for WT segmentation), across six segmentation schemes, for *13* correctly classified subjects (by a majority of segmentation schemes) was ${\text{HD}}_{\text{avg}}^{\text{WT}}=2.52\pm 0.22$ and for *18* misclassified subjects was ${\text{HD}}_{\text{avg}}^{\text{WT}}=5.92\pm 1.17$. Moreover, *8* (out of 16) subjects that were misclassified by all radiomic models had large segmentation errors (${\text{HD}}_{\text{avg}}^{\text{WT}}=7.09\pm 1.32$). This empirically demonstrated that a strong predictive performance on OS classification of brain gliomas requires accurate segmentation of brain tumor volume with small segmentation errors.

We also found that most subjects that failed on at least one radiomics model were short-term survivors (*8* subjects out of 31). Short-term survivors are typically associated with aggressive and heterogeneous tumor expressions (Beig et al., 2018) and, hence, one needs to augment the current feature set with appropriate measures of tumor heterogeneity for improved classification. Our analysis also revealed that the *WT* radiomics model, the *6-subregions* radiomics model, and the *21-subregions* radiomics model simultaneously misclassified *11* (out of *12*) subjects. This is attributed to the common requirement of accurate segmentation of WT volume for feature extraction and classification.

There are several limitations in our study as well. Of which, foremost is the limited dataset publicly available for an empirical study on OS classification tasks in brain gliomas, which is an often-encountered problem in clinical and translational imaging research. A large and balanced dataset would ideally help generalize the findings in this study to diverse tumor manifestations, gender, and demographics. While we employed shape, volumetric, and spatial features for radiomics-based prediction of OS in brain gliomas, augmenting the current feature set with more stable and predictive features and capturing tumor heterogeneity and aggressiveness may improve the classification of short-term survivors in brain gliomas. Combining the radiomics-based prediction of OS with explainable artificial intelligence (XAI) would be interesting as well. The five CNNs were trained using various combinations of Soft Dice and Cross Entropy loss functions. It would be interesting to see the impact of other loss functions, optimization schemes, and architectural engineering on segmentation accuracy and associated radiomic performance for OS classification in brain gliomas.

## Data Availability Statement

Publicly available datasets were analyzed in this study. The data can be downloaded from: https://www.med.upenn.edu/cbica/brats2020/data.html.

## Author Contributions

HMD designed the research. AS, UB, and HMD performed the research. STB and HMD performed the research on automatic segmentation. AS, STB, MN, and HMD wrote the codes and algorithms in Python. AS, UB, and HMD analyzed the data and results. AS, HMD, UB, and SB wrote the manuscript. HMD advised and mentored STB and MN. SB and HMD co-advised AS. All authors contributed to the article and approved the submitted version.

## Conflict of Interest

The authors declare that the research was conducted in the absence of any commercial or financial relationships that could be construed as a potential conflict of interest.

## Publisher’s Note

All claims expressed in this article are solely those of the authors and do not necessarily represent those of their affiliated organizations, or those of the publisher, the editors and the reviewers. Any product that may be evaluated in this article, or claim that may be made by its manufacturer, is not guaranteed or endorsed by the publisher.

## Footnotes

1 CBICA Image Processing Portal: https://ipp.cbica.upenn.edu/

2 N4-bias correction is not recommended for FLAIR sequence (Bakas et al., 2017c). To the best of our knowledge, no such behavior has been reported with T1 sequence. In our study, we used the T1 sequence for image registration and, hence, employed N4-bias correction without hesitation. Moreover, radiomic features (such as shape and volumetric features) were only extracted from segmentation masks.

3
https://pyradiomics.readthedocs.io

4
https://github.com/nipy/nibabel

5
https://github.com/FETS-AI/LabelFusion

## Appendix

**TABLE A1:** The 21 subregions defined by the Harvard-Oxford subcortical atlas are tabulated below.

Label	Anatomical Region
1	Left Cerebral White Matter
2	Left Cerebral Cortex
3	Left Lateral Ventricle
4	Left Thalamus
5	Left Caudate
6	Left Putamen
7	Left Pallidum
8	Brainstem
9	Left Hippocampus
10	Left Amygdala
11	Left Accumbens
12	Right Cerebral White Matter
13	Right Cerebral Cortex
14	Right Lateral Ventricle
15	Right Thalamus
16	Right Caudate
17	Right Putamen
18	Right Pallidum
19	Right Hippocampus
20	Right Amygdala
21	Right Accumbens
